# The association between albuminuria and coronary atherosclerotic burden in the general population

**DOI:** 10.1016/j.athplu.2026.100610

**Published:** 2026-09-21

**Authors:** Fredrik Bäckström Pegelow, Yi-Ting Lin, Anders Larsson, Andrei Malinovschi, Gunnar Engström, Johan Ärnlöv

**Affiliations:** a Center for Clinical Research, Region Dalarna, Falun, Sweden; b Department of Neurobiology, Care Sciences and Society, Division of Family Medicine and Primary Care, Karolinska Institute, Stockholm, Sweden; c Department of Family Medicine, Kaohsiung Medical University Hospital, Kaohsiung Medical University, Taiwan; d Department of Medical Sciences, Clinical Chemistry, Uppsala University, Uppsala, Sweden; e Department of Medical Sciences, Clinical Physiology, Uppsala University, Uppsala, Sweden; f Department of Clinical Sciences, Lund University, Malmö, Sweden; g School of Health and Welfare, Dalarna University, Falun, Sweden

**Keywords:** Albuminuria, Coronary atherosclerosis, Epidemiology, Coronary computed tomography angiography, Risk factors

## Abstract

**Background:**

Albuminuria predicts cardiovascular events, but its association with prevalent subclinical coronary atherosclerosis is less well defined. We examined the cross-sectional relationship between urinary albumin–creatinine ratio (UACR) and coronary atherosclerotic burden in middle-aged adults from Sweden nationwide.

**Methods:**

UACR was measured in fasting spot urine in 9424 SCAPIS participants from Uppsala and Malmö. Coronary atherosclerosis was quantified by coronary artery calcium score (CACS; n = 9277) and coronary computed tomography angiography (CCTA; n = 9187) for stenosis. Linear regression related log(UACR) to log(CACS) with sequential adjustment: Model 1 (age, sex, ethnicity, site), Model 2 (+blood pressure, antihypertensive treatment, diabetes and antidiabetic treatment), and Model 3 (+LDL cholesterol, lipid-lowering treatment, smoking status, and eGFR). Logistic regression examined prevalent calcification (CACS>0) and stenosis; subgroup analyses excluded CKD, cardiovascular disease, hypertension, and diabetes stepwise.

**Results:**

Higher UACR was associated with higher CACS in the full cohort: β = 0.21 (95% CI 0.14–0.27), 0.08 (0.01–0.15), and 0.07 (0.00–0.14) in Models 1–3, respectively. Associations persisted after excluding CKD and cardiovascular disease (n = 8448): β = 0.20 (0.13–0.27), 0.19 (0.12–0.26), and 0.07 (0.00–0.15). With further exclusions, Model 3 estimates attenuated and were non-significant (no CKD/cardiovascular disease/hypertension: β = 0.06 [−0.02 to 0.14]; additionally no diabetes: β = 0.05 [−0.03 to 0.14]). In Model 1, each log-increase in UACR was associated with higher odds of CACS>0 (OR 1.08, 95% CI 1.05–1.12) and stenosis (OR 1.04, 95% CI 1.01–1.08), but associations attenuated and were non-significant in Models 2–3.

**Conclusions:**

Albuminuria is associated with greater coronary calcification burden, including in broadly healthy individuals, supporting the concept that albuminuria may reflect vascular disease early in the cardiovascular disease continuum.

## Graphical abstract

**Figure:**
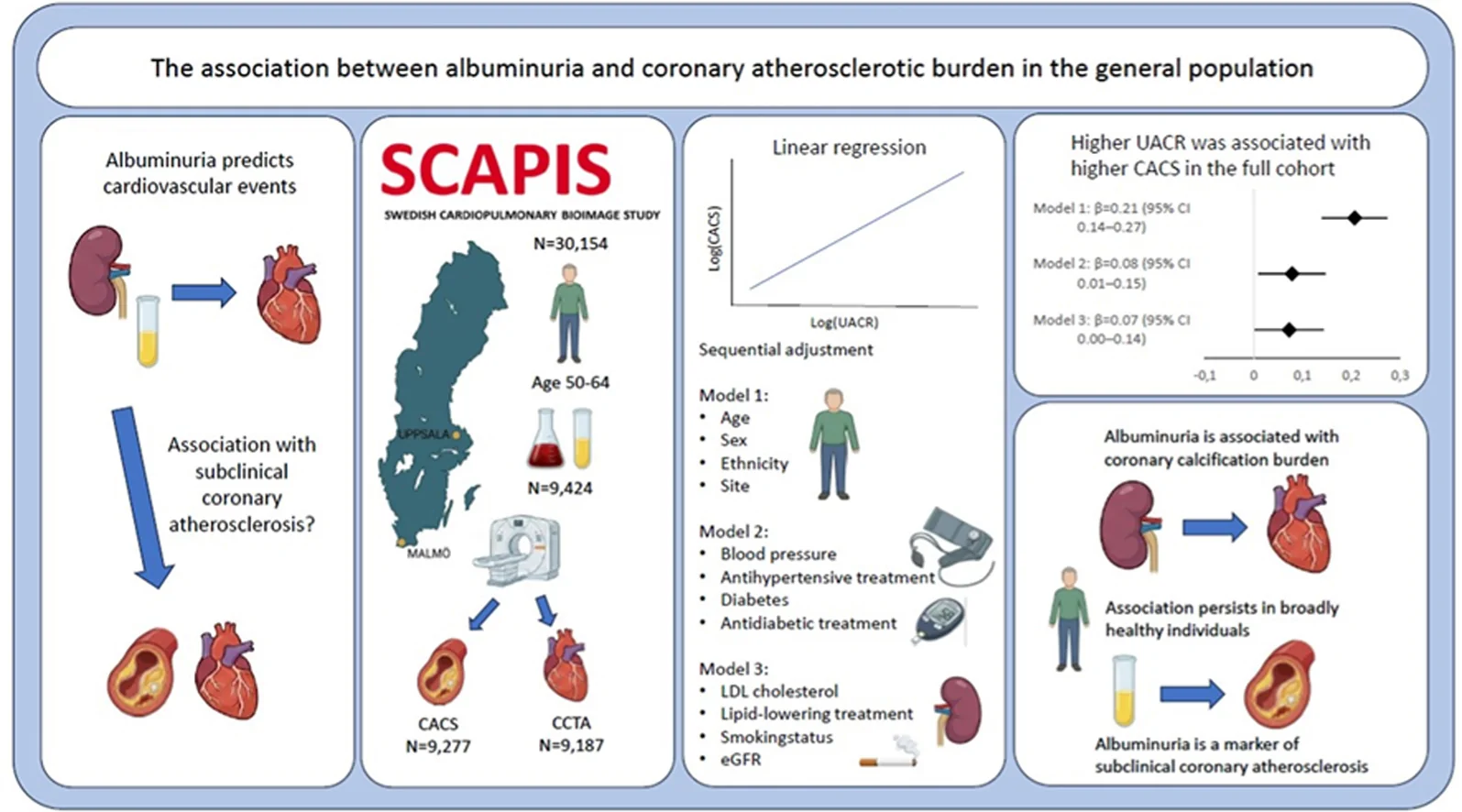

## 1 Introduction

Cardiovascular disease is the leading global cause of mortality and morbidity with ischemic heart disease being the largest contributor to the cardiovascular disease burden [1].

Asymptomatic coronary atherosclerosis is common in the general population and elevated coronary artery calcification convey a significant probability of substantial stenosis [2]. A recent Danish study reported that 46% of the individuals from the general population had subclinical coronary atherosclerosis and that those with subclinical obstructive coronary atherosclerosis had a more than 8-fold elevated risk for future myocardial infarction [3].

Albuminuria, a marker of kidney damage that reflects increased glomerular permeability to macromolecules and decreased tubular reabsorption [[4], [5], [6], [7]], is a common screening test in patients with hypertension or diabetes and used in the diagnosis and staging of kidney disease. Almost three decades ago the Steno hypothesis was formulated, proposing that albuminuria could serve as an indicator of cardiovascular risk, even in seemingly healthy individuals, as it mirrors both kidney-related vascular damage and systemic endothelial dysfunction [[8], [9], [10]]. Since then, it has been established that albuminuria is an important risk factor for incident cardiovascular disease in the general population, even in apparently healthy individuals without diabetes or hypertension [[11], [12], [13], [14], [15]]. It has been suggested that albuminuria screening and monitoring should be considered even in individuals with low risk of cardiovascular disease [12]. To what extent albuminuria is associated with prevalent asymptomatic coronary atherosclerosis is less known.

Therefore, we aimed to investigate the cross-sectional association between albuminuria and asymptomatic coronary atherosclerosis in the general population. Coronary artery atherosclerotic burden was assessed through state-of-the-art coronary computed tomography, including coronary artery calcium scoring (CACS) and the degree of coronary stenosis determined by coronary computed tomography angiography (CCTA). Prespecified subgroup analyses were conducted in individuals without known cardiovascular disease, kidney disease, hypertension, or diabetes.

We hypothesized that glomerular endothelial dysfunction, as indicated by low-grade albuminuria, also reflects atherosclerotic burden in larger arteries even in apparently healthy individuals.

## 2 Method

### 2.1 Study population

We utilized data from the Swedish CArdioPulmonary BioImage Study (SCAPIS) [16], a prospective observational study that recruited a total of 30,154 individuals aged 50-64. Participants were randomly selected from regions surrounding six university hospitals across Sweden between 2013 and 2018.

Each participant underwent a series of assessments, including blood and urine examinations, the collection of questionnaires, and computed tomography imaging during 2-3 visits to the study center. There was a possibility for each node to add on investigations to the core program, making use of the available extensive infrastructure. The baseline investigations were completed in 2018. For the present study we included participants from the Uppsala (n = 5036) and Malmö sites (n = 6251), where urinary albumin-creatinine ratio (UACR) was analyzed in addition to the core protocol. The whole SCAPIS study received approval from the ethical review board at Umeå University, Sweden (2010-228-31M), and the ethical boards at Uppsala University and Lund University, Sweden, approved the expanded study protocol at these study sites (EPN Uppsala University 2016/387 and 2018/315; Lund University 2016/1031).

At the first study visit, venous blood and spot urine samples were collected after an overnight fast of at least 8 h. The samples for biobanking were aliquoted and frozen at – 80 °C within 2 h from collection. The frozen urine samples were sent in site-specific batches to the University Hospital of Uppsala where urinary albumin was quantified using particle-enhanced immunological reagents from Abbott Laboratories (Abbott Park, IL, USA) on a BS-380 chemistry analyzer (Mindray, Shenzhen, China). Urinary creatinine levels were determined using an IDMS-calibrated enzymatic assay (Abbott Laboratories), also performed on the BS-380 (Mindray). From these measurements the urinary albumin-creatinine ratio (UACR) was calculated for each participant.

Coronary artery atherosclerosis was assessed through coronary computed tomography, including coronary artery calcium scoring (CACS) and the degree of coronary stenosis determined by coronary computed tomography angiography (CCTA). Details regarding cardiac imaging have previously been described [16]. In brief, total calcification levels in each artery was quantified using non-contrast enhanced images and summed to calculate a total CACS following international standards [17]. Stenosis severity based on CCTA was assessed visually by experienced radiologists and cardiologists across 18 coronary artery segments.

Participants with arterial stents or a history of CABG (Coronary Artery Bypass Graft) was classified as having prevalent stenosis. In cases where calcification evaluation was challenging (e.g. calcium blooming) the participant was also classified as having a stenosis.

### 2.2 Statistical analysis

All continuous baseline variables were reported using means (standard deviations) or medians (interquartile range) as appropriate, and categorical variables as n (%). In primary analyses we employed linear regression to examine the relationship between UACR (modelled as a continuous variable with natural logarithmic transformation to normalize for skewed distribution) and coronary calcification defined by CACS (continuous and log-transformed and with a small constant added to alleviate zero-inflation-issues). Covariates were grouped into three sequential models to evaluate the extent to which the association between UACR and coronary calcification was explained by demographic factors, major clinical determinants of albuminuria, established cardiovascular risk factors, and kidney function. Model 1 included demographic characteristics and study site. Model 2 additionally included blood pressure- and diabetes-related variables, as hypertension and diabetes are major determinants of albuminuria and established risk factors for coronary atherosclerosis. Model 3 further included additional established cardiovascular risk factors and eGFR to assess whether the association between UACR and coronary calcification persisted independently of these factors and overall kidney function. The models were specified as follows:

• **Model 1:** Multivariable linear regression model adjusted for age, sex, region of birth, and study site.
• **Model 2:** Model 1 with additional adjustment for systolic blood pressure, diastolic blood pressure, antihypertensive treatment, diagnosis of diabetes mellitus, and antidiabetic treatment.
• **Model 3:** Model 2 with additional adjustment for low-density lipoprotein cholesterol, lipid-lowering treatment, smoking status, and estimated glomerular filtration rate (eGFR).

Additionally, we performed multiple stratified analyses using step-by-step exclusion of individuals with chronic kidney disease (eGFR<60), known cardiovascular disease, hypertension or diabetes. We performed multiplicative interaction analyses to investigate if there was an effect modification by site.

In secondary analyses, we used logistic regression to examine the relationship between log-transformed UACR as a continuous variable and prevalent coronary calcification (CACS >0) versus no coronary calcification (CACS = 0) and the prevalence of coronary stenosis versus no coronary stenosis using CCTA data. These analyses provide additional insights if UACR may help identify individuals with prevalent atherosclerosis from those without.

## 3 Results

### 3.1 Baseline characteristics

Among the 9628 participants in the SCAPIS study who underwent additional urine examinations (4717 from Uppsala and 4911 from Malmö), 9424 had valid samples of urine albumin to urine creatinine ratio (UACR), as seen in Table 1. The mean age of participants was 57.7 years in Uppsala and 57.4 years in Malmö. The male population comprised 46.7% in Malmö and 48.7% in Uppsala. The proportion of current smokers was higher in Malmö (17.2%) than in Uppsala (8.9%), while more participants in Uppsala were never smokers (55.4% vs. 42.5%). Additionally, the coronary artery calcium score (CACS) was higher in Malmö (71.5) compared to Uppsala (53.5). In total, 9277 participants had complete CACS data, while 9187 participants had complete CCTA data from both Uppsala and Malmö.

**Table 1:** Baseline characteristics of participants categorized by enrollment site of the Swedish CArdioPulmonary bioImage Study (SCAPIS) cohort.

	Enrollment site	
	Malmö (N = 4911)	Uppsala (N = 4717)
Age, years*	57.4 (4.3)	57.7 (4.4)
Female, n (%)	2619 (53.3%)	2422 (51.3%)
Region of birth, n (%)		
Scandinavia	3849 (78.7%)	4233 (90.0%)
Europe	670 (13.7%)	183 (3.9%)
Asia	257 (5.3%)	189 (4.0%)
Other area	112 (2.3%)	97 (2.1%)
Smoking status, n (%)		
Never smoker	2089 (42.5%)	2612 (55.4%)
Former smoker	1852 (37.7%)	1445 (30.6%)
Current smoker	846 (17.2%)	421 (8.9%)
Diabetes Mellitus, n (%)	242 (5.1%)	197 (4.2%)
Body mass index*	27.3 (4.58)	27.0 (4.35)
Systolic blood pressure (mmHg)*	122 (16)	125 (16)
Diastolic blood pressure (mmHg)*	75 (10)	77 (10)
Plasma Glucose level (mg/dl)*	101 (23.3)	103 (18.2)
Albumin creatinine ratio (ug/mg)^†^	8.62 [3.32; 21.2]	2.91 [1.12; 10.7]
Anti-hyperlipidemia treatment, n (%)	403 (8.6%)	348 (7.4%)
Anti-diabetes treatment, n (%)	206 (4.4%)	159 (3.4%)
Anti-hypertensive treatment, n (%)	989 (20.1%)	868 (18.4%)
CCTA:		
Group 1 (no stenosis)	2780 (58.2%)	2638 (59.8%)
Group 2 (any level of stenosis)	1994 (41.8%)	1775 (40.2%)
Coronary artery calcium score (CACS) group, n (%)		
Group 1 (=0)	2666 (56.1%)	2806 (62.1%)
Group 2 (>0)	2086 (43.9%)	1714 (37.9%)
Coronary artery calcium score (CACS)*	71.5 (239)	53.5 (199)

CCTA: coronary computed tomography angiography.

CCTA group: 1: no stenosis/2: any level of stenosis or calcium blooming or stent or CABG.

CACS group: Group 1 (CACS = 0)/Group 2 (CACS>0).

Continuous variables are presented as *mean (standard deviation) or ^†^median [interquartile range] as appropriate.

### 3.2 Association between albuminuria and coronary atherosclerosis

In the primary analyses in the whole cohort there was a robust positive association between higher UACR and higher CACS scores in all multivariable models (Table 2). Associations also remained significant in Model 1 and 2, in stratified analyses when we excluded all individuals with chronic kidney disease (eGFR<60), known cardiovascular disease, hypertension and diabetes (Table 2). These associations were generally consistent in the stratified analyses after further adjustment for eGFR (Model 3) but slightly weaker and no longer significant in the healthiest strata (in participants without chronic kidney disease (eGFR>60), known cardiovascular disease, hypertension and diabetes.

**Table 2:** The association between urinary albumin-to-creatinine ratio and coronary artery calcification in the whole cohort and in subgroup analyses.

Participants	Model 1	Model 2	Model 3
	β-estimate (95% CI)	β-estimate (95% CI)	β-estimate (95% CI)
Whole cohort, N = 9277	0.21 (0.14, 0.27)***	0.08 (0.01, 0.15)*	0.07 (0.00, 0.14)*
No CKD, N = 9042	0.20 (0.13, 0.27)***	0.19 (0.12, 0.26)***	0.07 (0.00, 0.14)*
No CKD, no CVD. N = 8448	0.20 (0.13, 0.27)***	0.19 (0.12, 0.26)***	0.07 (0.00, 0.15)*
No CKD, no CVD, no hypertension. N = 6566	0.14 (0.06, 0.22)***	0.13 (0.05, 0.21)**	0.06 (−0.02, 0.14)
No CKD, no CVD, no hypertension, no diabetes. N = 6377	0.13 (0.04, 0.21)**	0.12 (0.03, 0.20)**	0.05 (−0.03, 0.14)

Multivariable linear regression portraying increase in log-transformed CACS per increase in logtransformed UACR. Multiple stratified analyses were also performed using step-by-step exclusion of individuals with CKD (eGFR>60), known CVD, hypertension or diabetes.

Model 1: Multivariable linear regression model with age, sex, ethnicity, and individual cohort (site) adjustments.

Model 2: Multivariable linear regression model adjusting for Model 1 covariates and additionally adjusting for systolic blood pressure, diastolic blood pressure, antihypertensive treatment, diagnosis of diabetes mellitus and antidiabetic treatment.

Model 3 (all cardiovascular risk factors and eGFR): Multivariable linear regression model with adjusting for Model 2 covariates and additionally adjusting for low-density lipoprotein, antihyperlipidemia treatment, smoking status and eGFR.

Abbreviations: CI: confidence interval, CACS: coronary artery calcification score, UACR: urinary albumin-to-creatinine ratio, eGFR: estimated glomerular filtration rate, CKD: chronic kidney disease, CVD: cardiovascular disease, ***: p < 0.001, **: p < 0.01, *: p < 0.05.

To examine the robustness of our findings given the differences in median UACR between study sites, we assessed potential effect modification by study site using multiplicative interaction terms between study site and log-transformed UACR in the full cohort. There was no evidence of effect modification by study site in any of the multivariable models (all p > 0.67). Consistently, the site-specific estimates were very similar in magnitude and direction; in Model 1, the regression estimates for the association between log-transformed UACR and log-transformed CACS were 0.21 (95% CI 0.12–0.30) in Uppsala and 0.20 (95% CI 0.10–0.30) in Malmö.

In secondary analyses, each log-increase in UACR was associated with 8% higher odds of prevalent coronary calcification and 4% higher odds of prevalent coronary stenosis in Model 1 (OR 1.08, 95% CI 1.05–1.12, p < 0.001 and OR 1.04, 95% CI 1.01–1.08, p = 0.01, respectively), but the associations were attenuated and no longer statistically significant in Models 2 and 3 (Table 3).

**Table 3:** The association between urinary albumin-to-creatinine ratio and prevalent coronary artery calcification and prevalent coronary stenosis respectively.

	Odds ratio	Low CI (2.5%)	High CI (97.5%)	P-value
	**CACS**			

Model 1	1.08	1.05	1.12	<0.01
Model 2	1.03	1.00	1.07	0.08
Model 3	1.03	0.99	1.06	0.14

	**CCTA**			

Model 1	1.04	1.01	1.08	0.01
Model 2	1.00	0.97	1.04	0.87
Model 3	1.00	0.97	1.03	0.89

Logistic regression on association between UACR as a continuous variable and prevalent coronary calcification (CACS >0) versus no coronary calcification (CACS = 0) or prevalence of coronary stenosis versus no coronary stenosis determined by CCTA.

Model 1: Multivariable linear regression model with age, sex, ethnicity, and individual cohort (site) adjustments.

Model 2: Multivariable linear regression model adjusting for Model 1 covariates and additionally adjusting for systolic blood pressure, diastolic blood pressure, antihypertensive treatment, diagnosis of diabetes mellitus and antidiabetic treatment.

Model 3 (all cardiovascular risk factors and eGFR): Multivariable linear regression model with adjusting for Model 2 covariates and additionally adjusting for low-density lipoprotein, antihyperlipidemia treatment, smoking status and eGFR.

Abbreviations: CI: confidence interval, UACR: urinary albumin-to-creatinine ratio, CACS: coronary artery calcification score, CCTA: coronary computed tomography angiography, eGFR: estimated glomerular filtration rate.

## 4 Discussion

### 4.1 Principal findings

In this study, we report a linear association between higher albuminuria (UACR) and higher burden of coronary artery calcification (CACS), independent of established cardiovascular risk factors and eGFR. This association was generally consistent in subgroup analyses in apparently healthy individuals without chronic kidney disease, cardiovascular disease, hypertension and diabetes, albeit not statistically significant in the full multivariable model in the healthiest strata. Moreover, we report that higher UACR was associated with a higher odds for both prevalent asymptomatic coronary artery calcification and stenosis (assessed by CCTA) in age and sex adjusted models. Taken together, these findings support the notion that albuminuria should not be regarded solely as a consequence of poorly controlled diabetes, hypertension or kidney disease but that higher albuminuria may also reflect an increased burden of coronary atherosclerosis also in the earliest stages of the cardiovascular continuum.

### 4.2 Comparison with the literature

In addition to being a well-established risk factor for incident cardiovascular events and mortality [[11], [12], [13], [14], [15]], a few community-based studies have previously reported associations between higher albuminuria and higher CACS [[18], [19], [20], [21]]. However, we are aware of only one previous study reporting these associations in individuals without diabetes, hypertension, cardiovascular disease and chronic kidney disease. In that study in approximately 1300 asymptomatic patients seeking health care in South Korea, microalbuminuria was associated with both coronary artery calcification assessed with CACS and a coronary stenosis >50% [22]. Thus, the present study, with an 8-fold larger study sample of healthy volunteers, confirms and extends these findings.

### 4.3 Potential mechanisms

The underlying mechanisms linking albuminuria to coronary atherosclerosis remain incompletely understood and it is unlikely that the presence of small amounts of albumin in the urine directly causes the increased cardiovascular risk. Elevated albuminuria is strongly associated with established risk factors for atherosclerosis [23,24]. The attenuation of the association between UACR and coronary calcification after progressive multivariable adjustment suggests that these shared cardiovascular risk factors account for part of the observed relationship. However, an association remained after adjustment for established cardiovascular risk factors and eGFR in the overall cohort and in individuals without known CKD or cardiovascular disease. This suggests that albuminuria may also reflect vascular processes not fully captured by conventional cardiovascular risk factors. One potential explanation is provided by the Steno-hypothesis that proposes that albuminuria is a marker for systemic endothelial dysfunction, a key factor in the development of atherosclerosis [[8], [9], [10]]. As such, albuminuria may be a more sensitive marker of subclinical vascular damage and atherosclerosis than other established risk factor such as high blood pressure, dyslipidemia and diabetes.

### 4.4 Clinical implications

We show that higher UACR was associated with a higher odds for prevalent coronary calcium (or prevalent coronary stenosis in age and sex adjusted models). Patients without known coronary artery disease but with high coronary artery calcification scores has been shown to have a risk for major adverse cardiac events similar to patients post myocardial infarction [25]. It has been shown that multifactorial intervention in patients with type 2 diabetes mellitus and microalbuminuria lower the risk of cardiovascular morbidity and mortality [26]. Furthermore, several pharmacological therapies that reduce albuminuria, including RAAS inhibitors and SGLT2 inhibitors, also reduce cardiovascular events, although these benefits are not necessarily solely mediated through effects on atherosclerotic disease [27]. Our findings extend these observations by demonstrating an association between UACR and subclinical coronary atherosclerotic burden also in individuals without known CKD or cardiovascular disease. This supports the concept that low-grade albuminuria may reflect vascular abnormalities already at an early stage of the cardiovascular disease continuum. Population-based studies have demonstrated that screening for albuminuria can identify a substantial proportion of individuals with previously unrecognized increased albuminuria [28]. However, the present cross-sectional study was not designed to determine whether UACR improves cardiovascular risk prediction or leads to clinically meaningful risk reclassification. Such questions should preferably be addressed in prospective studies using incident cardiovascular outcomes and established cardiovascular risk-prediction models. Our findings therefore provide further rationale for evaluating whether UACR may contribute to cardiovascular risk assessment beyond the patient groups in whom albuminuria is currently routinely assessed.

### 4.5 Strength and limitations

The main strength of this study is its contemporary cohort, which includes a detailed characterization of the participants, especially regarding risk factors for cardiovascular disease and state-of-the-art measurements of coronary calcification and stenosis with CACS and CCTA-measurements, respectively. An important limitation is that UACR, which is subject to considerable biological variability, was measured only once in each individual. Repeated measurements would have reduced the risk of misclassification, and the use of a single measurement may have attenuated the observed associations. Biological variability may also have contributed to the differences in median UACR observed between the two study sites, although differences in participant characteristics and potential site-related preanalytical or batch effects may also have played a role. Importantly, samples from both sites were analyzed at the same central laboratory using identical analytical methods. Both median UACR values were within the range of low-grade albuminuria generally observed in population-based cohorts. Furthermore, all analyses were adjusted for study site, and there was no evidence of effect modification by site in formal interaction analyses. Site-specific analyses likewise showed very similar associations between UACR and CACS. Taken together, these findings suggest that the between-site difference in absolute UACR levels did not materially affect the interpretation of the main findings. Nevertheless, residual site-related measurement differences cannot be completely excluded. Other limitations include the uncertain generalizability to other populations and potential residual confounding. The observational, cross-sectional design of the study prevents the establishment of causal relationships.

## 5 Conclusion

Albuminuria is associated with subclinical coronary atherosclerosis, even in apparently healthy individuals. These findings support the concept that albuminuria may reflect vascular disease early in the cardiovascular disease continuum. Additional studies are needed to determine whether UACR provides clinically useful predictive information beyond established cardiovascular risk factors.

## Ethics declaration

Written informed consent to take part in the study and to publish the article has been obtained from all participants or their legal representatives. The privacy rights of participants have been observed.

This study was performed in compliance with relevant laws, regulatory frameworks and guidelines where the research took place. The whole SCAPIS study received approval from the ethical review board at Umeå University, Sweden (2010-228-31M), and the ethical boards at Uppsala University and Lund University, Sweden, approved the expanded study protocol at these study sites (EPN Uppsala University 2016/387 and 2018/315; Lund University 2016/1031).

## Declaration of generative AI and AI-assisted technologies in the manuscript preparation process

During the preparation of this work, the authors used ChatGPT (OpenAI) to assist with language editing and to improve the clarity and readability of the manuscript. The authors reviewed and edited the output as needed and take full responsibility for the content of the published article.

## Sources of funding

The main funding body of The Swedish CArdioPulmonary bioImage Study (SCAPIS) is the 10.13039/501100003793Swedish Heart-Lung Foundation. The study is also funded by The 10.13039/501100004359Swedish Research Council and Center for Clinical Research, Region Dalarna.

## Conflict of interest

Johan Ärnlöv declares that he has received lecturing fees from AstraZeneca, Boehringer Ingelheim and Novartis and has served on advisory boards for AstraZeneca and Boehringer Ingelheim, unrelated to the present study. None of the other authors have any conflicts of interest to declare.
